# Characterization of Conserved and Promiscuous Human Rhinovirus CD4 T Cell Epitopes

**DOI:** 10.3390/cells10092294

**Published:** 2021-09-02

**Authors:** Marta Gomez-Perosanz, Tara Fiyouzi, Miguel Fernandez-Arquero, John Sidney, Alessandro Sette, Ellis L. Reinherz, Esther M. Lafuente, Pedro A. Reche

**Affiliations:** 1 Department of Immunology, School of Medicine, Complutense University of Madrid, 28040 Madrid, Spain; margom10@ucm.es (M.G.-P.); tarafiyo@ucm.es (T.F.); melafuente@med.ucm.es (E.M.L.); 2 Immunology Service, San Carlos University Hospital, 28040 Madrid, Spain; mferna01@ucm.es; 3 Division of Vaccine Discovery, La Jolla Institute for Immunology, La Jolla, CA 92037, USA; jsidney@lji.org (J.S.); alex@lji.org (A.S.); 4 Laboratory of Immunobiology, Dana-Farber Cancer Institute, Boston, MA 02215, USA; Ellis_Reinherz@dfci.harvard.edu

**Keywords:** Human rhinovirus, CD4 T cell, epitope, peptide

## Abstract

Human rhinovirus (RV) is the most common cause of upper respiratory infections and exacerbations of asthma. In this work, we selected 14 peptides (6 from RV A and 8 from RV C) encompassing potential CD4 T cell epitopes. Peptides were selected for being highly conserved in RV A and C serotypes and predicted to bind to multiple human leukocyte antigen class II (HLA II) molecules. We found positive T cell recall responses by interferon gamma (IFNγ)-ELISPOT assays to eight peptides, validating seven of them (three from RV A and four from RV C) as CD4 T cell epitopes through intracellular cytokine staining assays. Additionally, we verified their promiscuous binding to multiple HLA II molecules by quantitative binding assays. According to their experimental HLA II binding profile, the combination of all these seven epitopes could be recognized by >95% of the world population. We actually determined IFNγ responses to a pool encompassing these CD4 T cell epitopes by intracellular cytokine staining, finding positive responses in 29 out of 30 donors. The CD4 T cell epitopes identified in this study could be key to monitor RV infections and to develop peptide-based vaccines against most RV A and C serotypes.

## 1. Introduction

Human rhinovirus (RV) species A and C are the most frequent cause of viral respiratory tract infections worldwide [1]. In most individuals, RV infections are relatively mild and self-limited to the upper respiratory tract, being the most common cause of the common cold. However, in those patients with chronic respiratory diseases, in particular children and immunocompromised individuals, RVs can infect the lower respiratory tract, causing severe symptoms of bronchiolitis and pneumonia [2]. Likewise, it has been established that RV C species are the main cause of acute exacerbations in individuals with underlying chronic lung diseases, such as chronic obstructive pulmonary disease (COPD), cystic fibrosis, or asthma [3]. In fact, it is estimated that between 70–90% of asthma exacerbations requiring hospitalization in children are caused by RV C [4,5]. Currently, there is no vaccine for RV, and understating the immune response to RV is a necessary step.

As in any viral infection, adaptive immunity against RV is mediated by B and T cells, which recognize specific sites in antigens known as epitopes [6]. Identification of these RV-specific epitopes is of great interest for many reasons, including the monitoring of RV infections, understanding their immunopathology and the design of effective vaccines. Initial efforts to characterize the adaptive immune response to RV were aimed to identify B cell epitopes on VP1, VP2, and VP3 capsid proteins, which could be targeted by neutralizing antibodies [7]. Unfortunately, the RV genome is very plastic, and surface capsid proteins are particularly variable in different RV strains [8,9]. In fact, attending specifically to the variability found in these capsid proteins, there have been over 160–180 serologically distinct RV serotypes described [10]. As a result, neutralizing antibodies can only recognize serotype-specific epitopes, exhibiting little or no cross-reactivity between different serotypes [11,12]. Moreover, stimulating the humoral response as a means to provide effective protection against RV infection is difficult, requiring a high polyvalency (~25–50) to cover all possible RV strains [13].

Along with neutralizing antibodies, T cells also play a crucial role in the RV immunity [14], triggering both cellular and humoral immune responses. Interestingly, despite the low sequence identity among RV exposed capsid proteins, it has been shown that RV-specific CD4 and CD8 T cells can be reactive to multiple RV serotypes [15,16,17,18], revealing the presence of RV-specific conserved T cell epitopes. So far, only a few conserved RV-specific T epitopes have been identified [15,16,17,18,19]. A recent study [18] identified the first RV A and C conserved epitopes targeted by CD8 T cells, finding that they were distributed throughout the entire RV proteome, including proteins of the capsid and non-structural proteins. Given the link between antigen recognition by B and T cells, CD4 T cell epitope mapping has mainly focused on external RV capsid proteins VP1, VP2, and VP3 (reviewed in Stepanova et al. [20]), neglecting the remaining proteins.

In this work, we validated three RV A-specific CD4 T cell epitopes from VP4 capsid protein, along with four RV C-specific CD4 T cell epitopes mapping in various proteins of the capsid and in non-structural proteins. These epitopes are highly conserved in most RV A and C serotypes and exhibit a promiscuous binding to multiple human leukocyte antigen class II (HLA II) molecules that are highly frequent in the world population. In fact, a pool encompassing these epitopes was able to elicit interferon gamma (IFNγ) responses by CD4 T cells in 29 out of 30 donors. These identified CD4 T cell epitopes are clearly relevant for both monitoring RV infections and for designing an epitope-based vaccine against RV A and C serotypes with an ample population protection coverage.

## 2. Materials and Methods

### 2.1. Human Subjects

A study was carried out in 33 healthy adults aged 24–58 years (13 female and 20 males) without known allergies. All donors previously signed the informed consent document for the use of blood samples for research purposes, following the legislation corresponding to the Royal Decree-Law 1088/2005 of September 16 (reference number: BOE-A-2005-15514).

### 2.2. PBMCs Isolation and HLA Typing

Peripheral blood mononuclear cells (PBMCs) were isolated from peripheral blood samples (20 mL) by a density gradient on FicollPaque™ PLUS (Amersham, Darmstadt, Germany) following the manufacturer’s instructions. Genomic DNA was extracted from peripheral blood samples from 3 donors (1 female and 2 males) by using a MagNa pure compact instrument (Roche, London, UK), and HLA-DRB1, HLA-DQA1/B1, HLA-A and HLA-B typing were performed by PCR amplification and hybridization with allele-specific oligonucleotides, as described elsewhere [21].

### 2.3. Peptide Synthesis

Peptides were synthesized by ProteoGenix (Schiltigheim, France) at ≥95% purity as confirmed by reversed-phase high performance liquid chromatography (RP-HPLC). The mass of purchased synthetic peptides was verified by matrix-assisted laser desorption/ionization time-of-flight (MALDI-TOF) mass spectrometry (Research Assistance Center for Mass Spectroscopy at Complutense University of Madrid). Lyophilized peptides were dissolved in 40% dimethyl sulfoxide, diluted in ultra-pure water to a peptide concentration of 5 mM and stored at −80 °C until use.

### 2.4. CD4 T Cell Epitope Prediction

VP4 protein from RV A and whole consensus proteome from RV C were chosen as targets for CD4 T cell epitope prediction. The RV C consensus proteome was previously generated, as described by Gomez-Perosanz et al. [18], upon sequence variability analysis of 39 RV C full proteomes using the Shannon Entropy (H) as a variability metric. The RV A peptides were 18-mer long, overlapping by 10 amino acids, and covered the entire VP4 protein of RV A with accession number NP_042288.1. Peptide binding affinity to 20 HLA-DRB1 and 20 HLA-DQA1/B1 molecules was predicted using RANKPEP [22] and NETMHCII 2.3 servers [23]. HLA-DRB1 and HLA-DQA1/B1 molecules targeted for binding prediction are listed in Table S1. A peptide was considered to bind to a particular HLA II molecule if it ranked among the top 10% binding peptides with RANKPEP or NETMHCII. In addition, prediction of peptide binding to HLA II molecules with NETMHCII 2.3 was addressed, considering a range of sizes between 12–18 amino acids.

### 2.5. IFNγ-ELISPOT Assays

Peptide-specific IFNγ production by PBMCs from HLA II-typed donors was detected by standard IFNγ ELISPOT assays [24]. Briefly, 96-well PVDF plates (Millipore, Darmstadt, Germany) were coated with anti-IFNγ (1-D1K mAb, Mabtech, Stockholm, Sweden) and 1 × 10^5^ PBMCs were pleated with RPMI 1640 (Gibco, Waltham, MA, USA), supplemented with 10% of fetal bovine serum (Gibco, Waltham, MA, USA), 100 µg/mL streptomycin, 100 U/mL penicillin, and 2 mM L-glutamine (Lonza, Basel, Switzerland). Individual RV peptides were added at 10 μM. Plates were incubated at 37 °C and 5% CO_2_ and, after 24 h, were processed according to the manufacturer’s instructions [24]. PBMCs incubated with 10 μg/mL phytohemagglutinin (PHA) or without any stimuli were used as positive and background controls, respectively. The assay was run in triplicate for each individual peptide. The number of IFNγ-secreting cells (spot forming cells (SFCs)) was determined with an ELISPOT reader (ImmunoSpot 5.0, CTL Analyzers, Shaker Heights, OH, USA).

### 2.6. Intracellular Cytokine Staining

RV-specific T cells were expanded as described by Gomez-Perosanz et al. [18]. Briefly, donors’ PBMCs were cultured at a density of 2 × 10^6^ cells/mL in 24-well plates (BD Biosciences, Bedford, MA, USA) with RPMI 1640 (Gibco, Waltham, MA, USA), supplemented with 10% human serum (Gibco, Waltham, MA, USA), 100 µg/mL streptomycin, 100 U/mL penicillin, and 2 mM L-glutamine (Lonza, Basel, Switzerland). PBMCs were stimulated with individual RV peptides (10 µM) and 10 U/mL of IL-2 (Immunotools, Friesoythe, Germany). Cells were kept at 37 °C in 5% CO_2_ for 6 days, being fed and split as necessary with additional doses of peptide (10 µM) and IL-2 (10 U/mL) at day 3 of culture. In some experiments, the same PBMCs were also expanded and stimulated with 10 μM of RVC_1974–1990_ (GTSVFNTMINNIILRTL), RVA_2029–2037_ (YGDDVIFSY), CEFTA peptide pool (Mabtech, Stockholm, Sweden), CEF peptide pool (Mabtech, Stockholm, Sweden), RV-specific peptide pool, or an irrelevant peptide pool. RVC_1974–1990_ and RVA_2029–2037_ corresponded to RV-specific peptides that were not immunogenic in IFNγ ELISPOT assays. The CEFTA peptide pool (Mabtech, Stockholm, Sweden) contained 35 immunodominant HLA II-restricted epitopes from human cytomegalovirus (CMV), Epstein-Barr (EBV) and Influenza viruses and Tetanus toxin. The CEF peptide pool (Mabtech, Stockholm, Sweden) contained 23 HLA I-restricted immunodominant epitopes from human CMV, EBV and influenza viruses. The RV-specific peptide pool contained the 7 RV-specific CD4 T cell epitopes identified in this study. The irrelevant peptide pool contained four 15-mer peptides (VHNSQTFGRELPMYW, WCRSGYHPVMLNAQF, LRVKGCFNITMQPYD and FNWLRSEMCHKPVAY), randomly generated using RandSeq [25], which were predicted not to bind to any of the 20 HLA-DR or 20 HLA-DQ molecules selected in this study. All peptide pools were added at a final concentration of 10 μM of each peptide contained in the pool.

PBMCs expanded with the RV peptides were stimulated for 14 h, with 10 μM of each stimulus in the presence of Brefeldin A (5 μg/mL) (ThermoFisher Scientific, Waltham, MA, USA). Cells were washed with phosphate buffered saline (PBS) and surface stained with APC-conjugated anti-CD4 (REA623 mAb, Miltenyi Biotec, Somerville, MA, USA) and/or FITC-conjugated anti-CD8 (REA734 mAb, Miltenyi Biotec, Somerville, MA, USA) antibodies. The cell surface was fixed and permeabilized using the FoxP3 staining buffer set (eBioscience, San Diego, CA, USA), according to the manufacturer’s instructions, and then stained intracellularly with PE-conjugated anti-IFN-γ antibody (45–15 mAb, Miltenyi Biotec, Somerville, MA, USA). Stained cells were detected by flow cytometry (FACScalibur, BD Biosciences, Bedford, MA, USA).

### 2.7. Quantitative Binding Affinity Assays

Quantitative binding affinity of RV peptides to 8 different HLA-DRB1 molecules (DRB1*03:01, B1*07:01, B1*11:01, B1*11:04, B1*04:04, B1*08:02, B1*09:01 and B1*13:02) and 6 HLA-DQA1/B1 molecules (DQA1*01:01/B1*05:01, A1*01:02/B1*06:02, A1*02:01/B1*02:02, A1*03:01/B1*03:01, A1*05:01/B1*02:01, and A1*05:01/B1*03:01) was determined by classical competitive inhibition of binding assays, following the protocol described in Sidney et al. [26]. In brief, affinity-purified HLA II molecules (1–10 nM) were co-incubated at 37 °C or room temperature with 0.1–1 nM of a high-affinity binding radiolabeled peptide and the individual RV peptides in the presence of protease inhibitors. The respective high-affinity radiolabeled probe peptide used for each HLA II molecule was as listed by Sidney et al. [26]. Each unlabeled competitor RV peptide was tested at six different 10-fold concentrations (0.3 nM–30 µM) in three or more independent assays. After 2-day incubation, HLA II-peptide complexes were captured on anti-HLA II mAb-coated plates (L243 mAb for anti-HLA-DR and SPVL-3 mAb for anti-HLA-DQ) and the percentage of radioactivity was determined using the TopCount microscintillation counter (Packard Instrument Company, Downers Gorve, IL, USA). Finally, the concentration of the unlabeled peptide that inhibits the binding of the high-affinity radiolabeled peptide by 50% (IC_50_) was calculated.

### 2.8. Other Procedures

NetMHC 4.0 [27] was used for predicting peptides nested in RVA_65-82_ (IPTLQSPTVEACGYSDRI) binding to HLA I molecules. Epitope population protection coverage (PPC) was computed with the IEDB PPC tool [28], considering the allelic frequency of 21 different ethnicities around the world. Statistical analyses were performed using the statistical packages on GraphPad Prism 8 (GraphPad Software Inc., La Jolla, CA, USA). The non-parametrical Kruskal-Wallis test was applied to assess the statistical significance between means. Post-hoc analysis was performed using Dunn´s correction. and a *p* < 0.05 was considered statistically significant.

## 3. Results

### 3.1. Computational Selection of Conserved RV Peptides with Potential CD4 T Cell Epitopes

T cell epitope mapping is costly and time-consuming. Therefore, in order to reduce the experimental load, we followed two different approaches to predict and select CD4 T cell epitopes candidates. For RV A, we targeted the internal capsid protein VP4 for HLA II binding predictions, as it is highly conserved among different RV A serotypes [8,9,18]. Moreover, there are many copies of VP4 and it is located at the n-terminus of RV polyprotein, which both favors antigen processing and presentation to T cells [29]. However, since RV C is highly variable [18], for HLA II binding predictions, we targeted the entire consensus RV C proteome, with variable residues masked (generated previously by Gomez-Perosanz et al. [18]). Considering that CD4 T cells can only recognize peptides presented by HLA II molecules, we predicted CD4 T cell epitopes through HLA II binding predictions using RANKPEP and NetMHCII (details in Material and Methods). Since HLA II molecules are highly polymorphic and there are hundreds of allelic variants, we selected 20 HLA-DR and 20 HLA-DQ molecules that are common in the world population; they cover 99.8% of the population (listed in Table S1).

Following the criteria described in Materials and Methods, we finally selected 14 peptides (6 from RV A and 8 from RV C) for experimental scrutiny that contained potential CD4 T cell epitopes (Table 1). All of them were 12–18-mer long and were predicted to bind to at least one HLA-DRB1 and/or HLA-DQA1/B1molecule frequently expressed in the world population. We synthesized the peptides listed in Table 1 for experimental scrutiny and subjected them to functional assays.

### 3.2. Screening of CD4 T Cell Epitope Candidates by IFNγ-ELISPOT Assays

**Figure 1:**
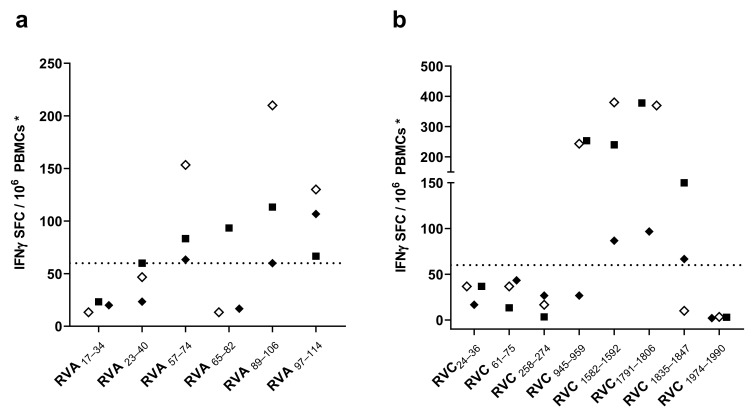
T cell responses to conserved rhinovirus (RV) A and C peptides. (**a**) Recall T cell responses to 18-mer overlapping peptides contained in VP4 protein from RV A. (**b**) Recall T cell responses to conserved 12–17-mer RV C peptides. Recall T cell responses were measured by interferon gamma (IFNγ)-ELISPOT assays in peripheral blood mononuclear cells (PBMCs) from three human leukocyte antigen class II (HLA-II) typed donors, as described in Materials and Methods. Results for each peptide are expressed as the mean of IFNγ spot forming cells (SFCs)/10^6^ PBMCs in each subject after subtracting the mean ± standard deviation of the background control. Each individual symbol represents results for the corresponding donor (Donor #1: empty diamond, Donor #2: filled square, Donor #3: filled diamond). The horizontal line represents the threshold used for positive responses (>60 SFC/10^6^ PBMCs). We found that 8 out of 14 peptides were able to elicit a positive IFNγ recall response in at least one donor.

We first screened for immunogenicity of the RV peptides by IFNγ-ELISPOT assays using PBMCs from three HLA II-typed donors stimulated with the individual RV peptides, as described in Materials and Methods. We considered a response to a peptide as positive if, after subtracting the mean ± standard deviation of the background control, the mean of detected IFNγ-SFC was at least 60 SFC/10^6^ PBMCs. As shown in Figure 1, 8 out of the 14 peptides (four of RV A and four of RV C) were able to trigger positive IFNγ recall responses (>60 SFC/10^6^ PBMCs) in at least one donor. Among RV A peptides (Figure 1a), RVA_89–106_, RVA_57–74_ and RVA_97–114_ prompted IFNγ recall response in all three donors, RVA_89–106_ being the peptide that promoted a greater IFNγ release. RVA_65–82_ only gave a positive result in Donor #2. Regarding RV C peptides (Figure 1b), all three donors responded to peptides RVC_1582–1592_ and RVC_1791–1805_, with a mean of 200–300 IFNγ-SFC per million PBMCs. The rest of the RV C peptides with a positive result (RVC_945–959_, RVC_1835–1847_) were able to stimulate IFNγ production by T cells in at least one donor. After IFNγ-ELISPOT assays, we discarded two RV A peptides and four RV C peptides that did not elicit enough IFNγ recall responses, selecting eight peptides to confirm peptide-specific IFNγ production by CD4 T cells.

### 3.3. Characterization of CD4 T Cell Epitopes

In order to validate the selected peptides as CD4 T cell epitopes, we first carried out intracellular cytokine staining assays to confirm that peptide-specific IFNγ production is mediated by CD4 T cells (details in Materials and Methods). Briefly, we first expanded PBMCs from each responding donor with the corresponding RV peptides (those producing a positive response in the previous section) and stained intracellularly to detect IFNγ positive CD4 T cells by flow cytometry.

**Figure 2:**
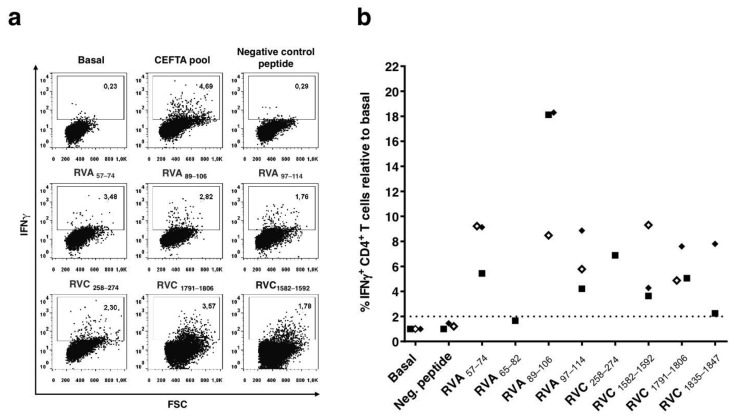
Peptide-specific interferon gamma (IFNγ) production by CD4 T cells in response to rhinovirus (RV) A and C conserved peptides. Donor peripheral blood mononuclear cells (PBMCs) expanded with the RV peptides were stimulated with individual peptides in the presence of Brefeldin A and stained intracellularly for detection of IFNγ production, as described in Materials and Methods. (**a**) Panel shows a representative experiment resulting from intracellular cytokine staining of PBMCs from Donor #2. Data is expressed as the percentage of peptide-specific IFNγ-producing CD4 T cells within the total of gated-CD4 T cells. (**b**) Plot depicting the percentage of peptide-specific IFNγ-producing CD4 T cells in responding donors. Represented values are relative to basal IFNγ-producing CD4 T cell in the absence of peptides. Individual symbols represent the mean of two independent experiments for each donor (Donor #1: empty diamond, Donor #2: filled square, Donor #3: filled diamond). The horizontal line represents the threshold used for positive responses. Positive and negative peptide controls were obtained by expanding and stimulating the same PBMCs with CEFTA peptide pool and the peptide RVC_1974–1990_ (GTSVFNTMINNIILRTL), respectively (see Materials and Methods). Peptide RVC_1974–1990_ was not immunogenic in IFNγ-ELISPOT assays.

As shown in Figure 2, IFNγ responses to RV peptides varied widely among different donors. We found peptide-specific CD4 T cells producing IFNγ in all responding donors to seven out of eight peptides (all but RVA_65–82_). The percentage of IFNγ-producing CD4 T cells actually increased 5–15 fold in all donors in response in any of those seven peptides. The strongest recall response was elicited by RVA_89–106_ (8–18 fold increase independently of the donor), followed by peptides RVA_57–74_, RVC_258–274_, RVC_1791–1806_, RVA_97–114_, RVA_1582–1592_, and RVC_1835–1847_.

Peptide RVA_65–82_ failed to stimulate IFNγ production by CD4 T cells from Donor #2 (<1.6% of IFNγ^+^ CD4^+^ T cells relative to basal), despite giving a positive response in ELISPOT assays in this donor. Thereby, we discarded this peptide as a CD4 T cell epitope. RVA_65–82_–specific IFNγ production detected by ELISPOT likely stems from shorter peptides released after processing of RVA_65-82_ that can be recognized by CD8 T cells. In fact, by flow cytometry, we could detect peptide-specific CD8 T cells producing IFNγ when stimulated with RVA_65–82_ (Figure S1). Moreover, we found two potential CD8 T cell epitopes in RVA_65–82_, peptides IPTLQSPTV and SPTVEACGY predicted to bind to HLA-B*35:03 (rank 1.5 and 0.40%, respectively), which is expressed by Donor #2. No other potential CD8 T cell epitope was predicted in RVA_65–82_ (IPTLQSPTVEACGYSDRI), judging by their ability to bind to the HLA I molecules expressed by Donor #2 (HLA-A*02:01, HLA-A*11:01, HLA-B*35:03, and HLA-B*44:03). The predicted binding of all 9-mer peptides in RVA_65-82_ to the HLA I molecules expressed by Donor #2 is provided in Table S2.

**Figure 3:**
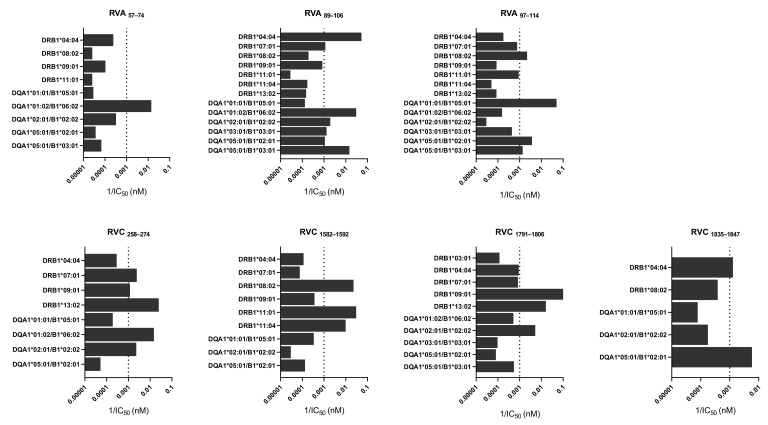
Binding affinity of rhinovirus (RV) peptides to human leukocyte antigen class II (HLA-II) molecules. The figure depicts the HLA-DR and -DQ molecules with detectable binding affinity for each of RV-specific CD4 T cell epitopes (IC_50_ < 40,000 nM). Binding affinity is given as 1/IC_50_ value (nM), as determined by competitive inhibition binding assays (details in Materials and Methods). Data is expressed as mean 1/IC_50_ obtained from three independent assays. The vertical line represents the threshold used as selection criteria for positive binding (IC_50_ < 1000 nM). Data used to generate the figure is provided in Table S3.

To further characterize the seven RV-specific CD4 T cell epitopes identified here, we determined the binding affinity of each RV peptide to 14 different HLA-DRB1 and HLA-DQA1/B1 molecules that are highly frequent in the world population (details in Materials and Methods). In Figure 3, we show the binding affinity of each individual RV peptide to all alleles that gave a detectable binding affinity (<40,000 nM in terms of IC_50_). The vast majority of experimentally verified CD4 T cell epitopes have an affinity for their corresponding HLA II restricting element of 1000 nM or better [30,31,32]. Therefore, we considered that peptides binding to a particular HLA II molecule with an IC_50_ <1000 nM IC_50_ could be potential CD4 T cell epitopes restricted by that HLA II element. Following this approach, we confirmed peptide binding to at least one HLA-DRB1 and/or HLA-DQA1/B1 molecule for all seven RV CD4 T cell epitopes. Peptide RVA_89–106_ (DSTITSQDVANAVVGYGV) was highly promiscuous, binding with high affinity (IC_50_ < 900 nM) to seven different HLA II molecules. Altogether, the seven RV CD4 T cell epitopes could bind to seven different HLA-DR (HLA-B1*04:04, B1 DRB1*07:01, B1*08:02, B1*09:01, B1*11:01, B1*11:04, and B1*13:02) and 6 HLA-DQ molecules (HLA-DQA1*01:01/B1*05:01, A1*01:02/B1*06:02, A1*02:01/B1*02:02, A1*03:01/B1*03:01, A1*05:01/B1*02:01 and A1*05:01/B1*03:01).

### 3.4. Population Coverage of RV-Specific T Cell Responses

**Figure 4:**
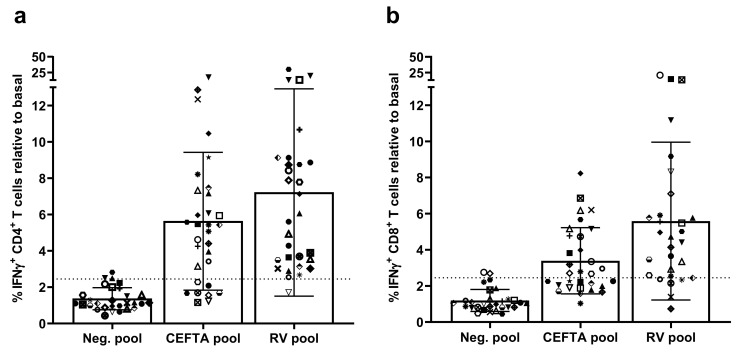
T cell responses to the rhinovirus (RV)-specific CD4 T cell epitope pool. Interferon gamma (IFNγ)-production by CD4 (**a**) and CD8 (**b**) T cells in response to different peptide pools. Peripheral blood mononuclear cells (PBMCs) from 30 donors were expanded and stimulated with a peptide pool containing 10 μM of each RV-specific CD4 T cell epitope identified in this study in the presence of Brefeldin A, labeled with anti-CD4 and anti-CD8 antibodies and stained intracellularly for IFNγ (see Materials and Methods). Results are expressed as the percentage of peptide-specific IFNγ-producing CD4 or CD8 T cells within the total of gated-CD4 or CD8 T cells as relative to the basal IFNγ production of each donor. Basal IFNγ production was obtained by incubating donor PBMCs without the addition of exogenous peptide. The CEFTA peptide pool was used as a positive control, and as a negative control, we used a peptide pool containing four irrelevant peptides (see Materials and Methods). Each individual symbol represents a different donor. Mean and standard deviation of all donors tested are plotted. The vertical line represents the threshold used as selection criteria for positive responses (>2.5% IFNγ^+^ CD4^+^ or CD8^+^ T cells fold increase as relative to basal).

According to their HLA II binding profiles, the seven epitopes identified in this work could be expected to elicit responses in up to 98% of the population, regardless of their ethnicity [28] (see Materials and Methods). This estimation is computed after the assumption that CD4 T cell epitopes shown to be immunogenic under a particular HLA II context will also be immunogenic in any subject expressing at least one of the HLA II molecules confirmed to bind the epitopes. There is considerable evidence for this assumption [33,34,35], and we tested its extent. To that end, we determined recall CD4 T cell responses to a pool encompassing all seven CD4 T cell epitopes in 30 donors through intracellular IFNγ staining, as described earlier. We confirmed positive CD4 T cell responses in 29 out of 30 subjects: 96.6% of the cohort (Figure 4).

The magnitude of the response depended on the donor, varying from a 1.71- to a 30.03-fold increase of IFNγ-producing CD4 T cells in response to the peptides, but, on average, increased 7.17-fold (Figure 4a). Interestingly, the CD4 T cell recall response to the RV-specific peptide pool was greater than that to the commercial CEFTA peptide pool (% IFNγ^+^ CD4^+^ T cells average fold increase of 7.17 and 5.62, respectively), although not in a statistically significant manner (*p* = 0.291). Moreover, more subjects responded to the RV-specific peptide pool (96.6%) than the CEFTA peptide pool (73.3%). We considered a donor responding to the RV-specific peptide pool if the percentage of IFNγ^+^ CD4^+^ T cells was at least 2.5 times greater than the basal IFNγ response of the donor.

Given that CD4 T cell epitopes can bear nested CD8 T cell epitopes [18], which are key for antiviral responses, we also tested CD8 T cell responses to the RV-specific peptide pool. We could detect an increase of IFNγ-producing CD8 T cells in response to the RV peptide pool in 24 out of 30 donors (80.0%) (Figure 4b). Exclusively considering the responses in the responding subjects, the average CD8 T cell response was lower than that determined for CD4 T cells (average fold increase in % IFNγ^+^ CD4^+^ and CD8^+^ T cells was 6.47 and 7.38, respectively).

Together, these results reveal that the seven conserved RV-specific CD4 T cell epitopes identified in this work can activate both, RV-specific CD4 and CD8 T cells, providing a broad population coverage.

## 4. Discussion

Human rhinoviruses (RVs) are considered to be mild pathogens, and RV infections are often downplayed. However, RV infections are a leading cause behind severe bronchiolitis and are also linked to acute exacerbations of chronic pulmonary diseases [2]. Moreover, RV infections have a large economic impact worldwide in terms of healthcare costs and work absenteeism [36]. Thereby, there is a great interest in developing effective therapeutic and prophylactic interventions against RV. Given that RVs possess positive-sense single-stranded RNA genomes, a promising therapeutic approach will be the use of DNAzymes, consisting of antisense single-stranded DNA molecules, which specifically bind and degrade RNA target molecules through enzymatic cleavage [37,38]. However, developing a vaccine would be the most cost-effective approach to combat RV infections.

RVs are small RNA viruses (~7500 bp) belonging to the family Picornaviridae and the genus Enterovirus. RVs exhibit highly variable genomes and have been classified in three species: RV A, B, and C. RV A and C are the more relevant species in the clinic as they are responsible for more than 90% of RV infections [1,10]. RVs are known to induce potent humoral and cellular immune responses [7]. The humoral response includes IgG and IgA neutralizing antibodies directed against viral surface proteins, which can provide protective immunity to secondary infections with related strains [11]. RV-specific T cell responses are particularly relevant to contain RV infection and clear viral particles. In healthy individuals, RV infections induce a T-helper type 1 (Th1) polarization of CD4 T cells, which contributes to activation of cytotoxic CD8 T cells capable of killing infected cells. However, in asthmatic individuals, RV infections have been shown to induce increased levels of interleukin (IL)-4 and IL-13, along with an increased infiltration of macrophages and neutrophils on the respiratory tract, which is linked to the ability of RV to enhance asthma exacerbations [39,40]. Most studies of RV immunity have focused on the characterization of antigen targets for antibodies, which have allowed to classify RVs into different serotypes; so far, more than 180 distinct RV serotypes have been identified [10]. However, the study of the targets of RV-specific T cell responses has received less attention. In fact, we recently described the first RV-specific CD8 T cell epitopes reported to date [18]. Since antigen recognition by CD4 T cells and B cells is linked, there are more known CD4 T cell antigen targets. Thus, some RV-specific CD4 T cell epitopes have been mainly identified on capsid proteins targeted also by antibody responses [15,16,17,19]. In this study, we identified novel RV-specific CD4 T cell epitopes through a computer-aided approach.

Identification of CD4 T cell epitopes generally begins with the selection of peptides suitable for presentation by HLA II molecules [41]. However, HLA II molecules are extremely polymorphic, and allelic variants bind and present distinct sets of peptides [42]. Thus, we targeted RV for peptide binding predictions to 20 HLA-DR and 20 HLA-DQ molecules, which have a combined phenotypic frequency of 99.80% in the world population [28]. We selected 14 peptides for experimental scrutiny after HLA II binding predictions (Table 1). In the end, we could validate trough IFNγ-ELISPOT assays (Figure 1) and intracellular cytokine staining (Figure 2) three RV A-specific and four RV C-specific CD4 T cell epitopes mapping in various proteins of the capsid and in non-structural proteins (Summarized in Table 2). These CD4 T cell epitopes are highly conserved, can bind to various HLA II molecules (Figure 3), and none of them have been previously reported. All three RV A-specific epitopes (RVA_57–74_, RVA_89–106_, and RVA_97–114_) are located on the internal capsid protein VP4. VP4 is located on the amino terminal extreme of the RV polyprotein, and this location has been shown to favor antigen processing and presentation to T cells [29]. Of the four RV C-specific epitopes, one (RVC_258–274_) is located in VP2 capsid protein. The other three RV C-specific epitopes (RVC_1582–1592_, RVC_1791–1806_, and RVC_1835–1847_) are distributed on viral proteins 3A and 3D, which are both implicated in the replication and assembly of the viral genome [8]. To our knowledge, these are the first CD4 T cell epitopes identified in these RV proteins.

The CD4 T cell epitopes identified in this work were capable of inducing strong IFNγ recall responses (Figure 2) and could bind to various HLA II molecules (Figure 3). By comparing the HLA II-typing of the responding donors with the experimental HLA II binding profile of the epitopes (Table S4), we could anticipate the potential restriction elements of some epitopes. Thus, RVC_1582-1592_ could be restricted by HLA-DRB1*11:01 since it could bind RVC_1582–1592_ with high affinity, and the relevant allele is expressed by the responding donors (Donors #2 and #3). In a similar way, HLA-DRB1*07:01 could restrict the response of peptide RVC_258–274_ and DQA1*05:01/B1*03:01 could restrict the response of peptide RVA_97–114_. However, we did not verify formally that these were the restrictions elements responsible for the response. For some of the epitopes (RVA_57–74_ and RVC_1791–1806_), the HLA II-typing of the responding donors did not match any of the HLA II molecules that could bind the epitopes. These epitopes must surely be presented to CD4 T cells by other HLA II molecules expressed by the donor that were not tested in the binding assays. It is worth noting that predicted peptide binding to HLA II molecules could be confirmed experimentally for 94% of peptide-HLA II pairs. However, many of the HLA II molecules that did experimentally bind peptides with an IC_50_ < 1000 nM (12 of 25) could not be predicted.

Epitopes shown to be immunogenic in the HLA II context of a particular subject are also expected to be immunogenic in other subjects, provided that they express any of the HLA II molecules that such an epitope can bind [33,34,35]. Under this assumption, and taking into consideration the HLA II binding profiles, our peptides were expected to elicit responses in up to 98% of the population, regardless of their ethnicity [28]. A pool encompassing these epitopes was indeed able to elicit IFNγ recall responses by CD4 T cells in 29 out of 30 donors (96.6%) (Figure 4a). We realize that all donors were Caucasians, but we detected more responses to this pool than a commercial peptide pool including 35 peptides (CEFTA pool), often used as a positive control for ELISPOT assays. Detection of peptide-specific IFNγ recall responses by CD4 T cells reveals that these peptides are bona fide CD4 RV-specific T cell epitopes, which are processed and targeted during RV infections. Interestingly, we could likewise detect strong IFNγ recall responses by CD8 T cells in 80% of the donors (Figure 4b). CD8 T cell responses to CD4 T cell epitope are somewhat expected, since CD4 T cell epitopes can bear shorter peptides (9–11-mer) capable of binding to HLA I molecules and can be recognized by CD8 T cells. In fact, in a previous work, we identified CD8 T cell epitopes within some of the CD4 T cell epitopes reported here [18]. Moreover, we also described that RVC_1791–1806_ (GLEPLDLNTSAGFPYV) is an unusually long HLA-A*02:01-restricted CD8 T cell epitope [18].

Given the extent of the responses, the three RV A- and four RV C-specific CD4 T cell epitopes identified are of particular interest for monitoring RV infections. More importantly, these epitopes represent excellent candidates to develop an epitope-based vaccine against RV, as they can also induce CD8 T cell responses. Moreover, they could be combined with additional CD8 T cell epitopes to enhance such responses. It is noteworthy that CD4 T cell epitopes alone or in conjunction with CD8 T cell epitopes could be readily incorporated into RNA vaccine technologies for parenteral or intranasal forms of administration in the future.

## Figures and Tables

**Table 1:** Predicted HLA II-binding profile of the RV A and C peptides selected in this study.

Virus	Peptide	Sequence	Protein ^1^	Position ^2^	Predicted HLA II Binding Profile ^3^	
					HLA-DRB1	HLA-DQA1/B1
RV A	RVA_17–34_	NSVSNGSSLNYFNINYFK	VP4	17–34	DRB1*12:01	DQA1*01:01/B1*05:01
	RVA_23–40_	LNYFNINYFKDAASSGAS	VP4	23–40	DRB1*04:01 DRB1*04:04 DRB1*08:02 DRB1*12:01	DQA1*01:01/B1*05:01 DQA1*01:01/B1*05:02 DQA1*01:04/B1*05:03
	RVA_57–74_	VKDVLEKGIPTLQSPTVE	VP4	57–74	DRB1*11:01	-
	RVA_65–82_	IPTLQSPTVEACGYSDRI	VP4	65–82	-	DQA1*05:01/B1*03:02
	RVA_89–106_	DSTITSQDVANAVVGYGV	VP4	89–106	-	DQA1*01:02/B1*06:02 DQA1*02:01/B1*02:02 DQA1*03:01/B1*03:01 DQA1*05:01/B1*03:01
	RVA_97–114_	VANAVVGYGVWPHYLTPE	VP4	97–114	DRB1*04:04	DQA1*01:01/B1*05:01 DQA1*03:01/B1*03:01 DQA1*05:01/B1*03:01 DQA1*05:01/B1*04:02 DQA1*06:01/B1*04:02
RV C	RVC_24–36_	VVKYFNINYYKDA	VP4	24–36	DRB1*12:01 DRB1*15:01	DQA1*01:01/B1*05:01 DQA1*01:02/B1*05:02
	RVC_61–75_	LTNPALMSPSVEACG	VP4	61–75	-	DQA1*01:03/B1*06:03 DQA1*02:01/B1*03:03 DQA1*05:01/B1*03:02 DQA1*05:01/B1*03:03
	RVC_258–274_	INLRTNNSSTIVVPYIN	VP2	258–274	DRB1*13:02	DQA1*01:02/B1*05:01 DQA1*01:02/B1*06:02 DQA1*01:03/B1*06:03 DQA1*02:01/B1*03:03 DQA1*05:01/B1*03:03
	RVC_945–959_	YEIQESEYYPKHIQY	2A	945–959	-	DQA1*01:04/B1*05:03
	RVC_1582–1592_	KEKFRDIRRFIP	3A	1582–1592	DRB1*08:02 DRB1*11:01	-
	RVC_1791–1806_	GLEPLDLNTSAGFPYV	3D	1791–1806	DRB1*07:01 DRB1*09:01 DRB1*13:02	-
	RVC_1835–1847_	DLPYVTYLKDELR	3D	1835–1847	-	DQA1*02:01/B1*02:02 DQA1*05:01/B1*02:01
	RVC_1974–1990_	GTSVFNTMINNIILRTL	3D	1974–1990	DRB1*01:01 DRB1*01:03 DRB1*04:01 DRB1*04:03 DRB1*04:04 DRB1*04:05 DRB1*07:01 DRB1*13:02	DQA1*01:02/B1*05:01

^1^ Protein of RV that contains the peptide sequence. ^2^ Position of the peptide in the selected reference RV polyproteins. ^3^ HLA II molecules predicted to bind the corresponding peptides.

**Table 2:** Summary of the RV-specific CD4 T cell epitopes identified in this study.

Peptide	Sequence	Protein	Confirmed HLA II Binding Profile ^1^		PPC ^2^
			HLA-DRB1	HLA-DQA1/B1	
RVA_57–74_	VKDVLEKGIPTLQSPTVE	VP4	-	DQA1*01:02/B1*06:02	34.55
RVA_89–106_	DSTITSQDVANAVVGYGV	VP4	DRB1*04:04 DRB1*07:01	DQA1*01:02/B1*06:02 DQA1*02:01/B1*02:02 DQA1*03:01/B1*03:01 DQA1*05:01/B1*02:01 DQA1*05:01/B1*03:01	95.09
RVA_97–114_	VANAVVGYGVWPHYLTPE	VP4	DRB1*08:02	DQA1*01:01/B1*05:01 DQA1*05:01/B1*02:01 DQA1*05:01/B1*03:01	81.58
RVC_258–274_	INLRTNNSSTIVVPYIN	VP2	DRB1*07:01 DRB1*09:01 DRB1*13:02	DQA1*01:02/B1*06:02 DQA1*02:01/B1*02:02	67.68
RVC_1582–1592_	KEKFRDIRRFIP	3A	DRB1*08:02 DRB1*11:01 DRB1*11:04	-	17.37
RVC_1791–1806_	GLEPLDLNTSAGFPYV	3D	DRB1*09:01 DRB1*13:02	DQA1*02:01/B1*02:02	36.28
RVC_1835–1847_	DLPYVTYLKDELR	3D	DRB1*04:04	DQA1*05:01/B1*02:01	52.04

^1^ HLA II molecule binding the peptide with a high binding affinity (IC_50_ < 1000 nM), as determined by competitive inhibition binding assays. ^2^ Population protection coverage (PPC), meaning the percentage of the world population that expresses at least one of the HLA II alleles. PPC was computed using the IEDB PPC tool [28] considering the allelic frequency of 21 different ethnicities around the world. The PPC of all seven epitopes reaches 98%.

## Data Availability

The data presented in this study are contained within the article and/or supplementary material and are also available on request from the corresponding author.

## Supplementary Materials

The following are available online at https://www.mdpi.com/article/10.3390/cells10092294/s1. Table S1: HLA II molecules used for predicting peptide binding; Table S2: HLA I binding predictions of all 9-mer peptides contained in RVA_65-82_ to HLA I molecules expressed by Donor #2; Table S3: Binding affinity data of RV peptides to HLA II molecules; Table S4: IFNγ-responses from HLA II-typed donors to RV-specific CD4 T cell epitopes; Figure S1: Peptide-specific IFNγ production by CD8 T cells.
