# Supplementary material for: Characterization of Conserved and Promiscuous Human Rhinovirus CD4 T Cell Epitopes
Source: Cells. 2021 Sep 2;10(9):2294. doi: 10.3390/cells10092294 (PMC8471592; doi:10.3390/cells10092294)
Supplement: Supplementary file 1 [file cells-10-02294-s001.zip › cells-1341828-supplementary/Figure S1.pdf]

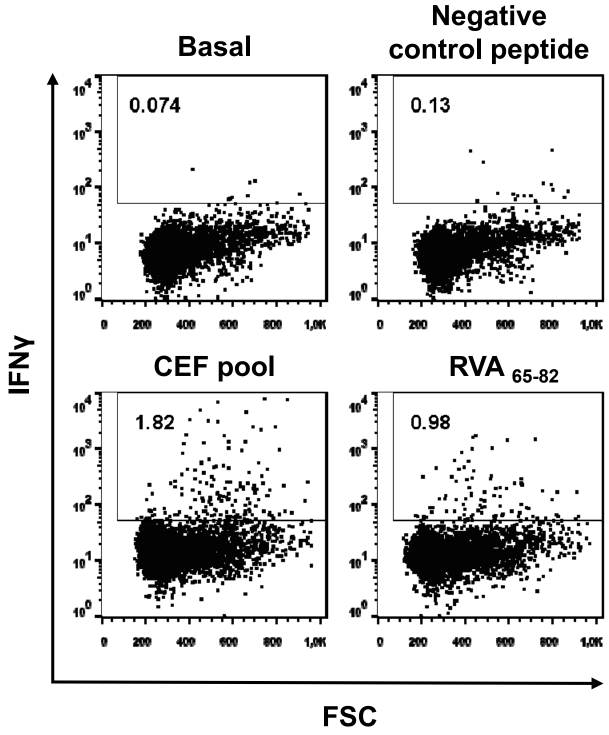

Figure S1. Peptide-specific IFNγ production by CD8 T cells. PBMCs from Donor #2 previously expanded (see Materials and Methods) were stimulated with 10μM of $HRVA_{65-82}$ peptide in the presence of Brefeldin A, labeled with anti-CD8 antibody and stained intracellularly for IFNγ. Data is expressed as the percentage of IFNγ-producing CD8 T cells within the total of gated-CD8 T cells. Basal IFNγ production was obtained by incubating donor PBMCs without the addition of exogenous peptides. CEF peptide pool was used as positive control and the irrelevant peptide $RVA_{2029-2037}$ (YGDDVIFSY) as negative control.
