# Supplementary material for: Characterization of Conserved and Promiscuous Human Rhinovirus CD4 T Cell Epitopes
Source: Cells. 2021 Sep 2;10(9):2294. doi: 10.3390/cells10092294 (PMC8471592; doi:10.3390/cells10092294)
Supplement: Supplementary file 1 [file cells-10-02294-s001.zip › cells-1341828-supplementary/Table S1.pdf]

**Table S1.** HLA II molecules used for predicting peptide binding.

| HLA-DRB1 | | HLA-DQA1/DQB1 | |
|---|---|---|---|
| **Molecule** | **Prediction Method** | **Molecule** | **Prediction Method** |
| DRB1*01:01 | RANKPEP and NETMHCII | DQA1*01:01/B1*05:01 | RANKPEP and NETMHCII |
| DRB1*01:03 | NETMHCII | DQA1*01:02/B1*05:01 | NETMHCII |
| DRB1*03:01 | RANKPEP and NETMHCII | DQA1*01:02/B1*05:02 | NETMHCII |
| DRB1*04:01 | RANKPEP and NETMHCII | DQA1*01:02/B1*06:02 | RANKPEP and NETMHCII |
| DRB1*04:02 | RANKPEP and NETMHCII | DQA1*01:03/B1*06:03 | NETMHCII |
| DRB1*04:03 | NETMHCII | DQA1*01:04/B1*05:03 | NETMHCII |
| DRB1*04:04 | RANKPEP and NETMHCII | DQA1*02:01/B1*02:02 | NETMHCII |
| DRB1*04:05 | RANKPEP and NETMHCII | DQA1*02:01/B1*03:01 | NETMHCII |
| DRB1*07:01 | RANKPEP and NETMHCII | DQA1*02:01/B1*03:03 | NETMHCII |
| DRB1*08:01 | RANKPEP and NETMHCII | DQA1*02:01/B1*04:02 | NETMHCII |
| DRB1*08:02 | NETMHCII | DQA1*03:01/B1*03:01 | NETMHCII |
| DRB1*09:01 | RANKPEP and NETMHCII | DQA1*03:01/B1*03:02 | RANKPEP and NETMHCII |
| DRB1*10:01 | NETMHCII | DQA1*03:03/B1*04:02 | NETMHCII |
| DRB1*11:01 | RANKPEP and NETMHCII | DQA1*04:01/B1*04:02 | NETMHCII |
| DRB1*11:04 | RANKPEP and NETMHCII | DQA1*05:01/B1*02:01 | RANKPEP and NETMHCII |
| DRB1*13:01 | RANKPEP and NETMHCII | DQA1*05:01/B1*03:01 | NETMHCII |
| DRB1*13:02 | RANKPEP and NETMHCII | DQA1*05:01/B1*03:02 | NETMHCII |
| DRB1*15:01 | RANKPEP and NETMHCII | DQA1*05:01/B1*03:03 | NETMHCII |
| DRB1*15:02 | NETMHCII | DQA1*05:01/B1*04:02 | NETMHCII |
| DRB1*16:02 | NETMHCII | DQA1*06:01/B1*04:02 | NETMHCII |
