# Supplementary material for: Characterization of Conserved and Promiscuous Human Rhinovirus CD4 T Cell Epitopes
Source: Cells. 2021 Sep 2;10(9):2294. doi: 10.3390/cells10092294 (PMC8471592; doi:10.3390/cells10092294)
Supplement: Supplementary file 1 [file cells-10-02294-s001.zip › cells-1341828-supplementary/Table S2.pdf]

**Table S2.** HLA I binding predictions of all 9-mer peptides contained in $RVA_{65\text{-}82}$ to HLA I molecules expressed by Donor #2.

| Peptide | HLA-A*02:01 | | HLA-A*11:01 | | HLA-B*35:03 | | HLA-B*44:03 | |
|---|---|---|---|---|---|---|---|---|
| | nM [1] | Rank [2] | nM [1] | Rank [2] | nM [1] | Rank [2] | nM [1] | Rank [2] |
| IPTLQSPTV | 25527.8 | 37 | 36740.8 | 55 | 20083.4 | 1.50 | 45948.5 | 75 |
| PTLQSPTVE | 43202.2 | 95 | 36195.5 | 55 | 45978.8 | 42 | 47291.1 | 90 |
| TLQSPTVEA | 1000.5 | 12 | 14118.8 | 12 | 42224.8 | 18 | 46461.4 | 80 |
| LQSPTVEAC | 15383.3 | 21 | 28840.9 | 30 | 44072.8 | 26 | 37641.3 | 28 |
| QSPTVEACG | 39107.6 | 80 | 36351.3 | 55 | 42102.1 | 17 | 47400.8 | 95 |
| SPTVEACGY | 34513.8 | 60 | 26120.1 | 24 | 8610.6 | 0.40 | 18861.2 | 13 |
| PTVEACGYS | 37036.1 | 70 | 35701.5 | 55 | 46555.0 | 50 | 47916.4 | 99 |
| TVEACGYSD | 36896.5 | 70 | 25081.5 | 23 | 44650.7 | 30 | 43840.2 | 55 |
| VEACGYSDR | 41959.7 | 90 | 22699.0 | 19 | 46345.9 | 47 | 13373.2 | 11 |
| EACGYSDRI | 31090.2 | 50 | 35409.5 | 50 | 44923.0 | 32 | 34618.1 | 22 |

[1] Predicted binding affinity for the corresponding allele. [2] Peptide rank (%) to a set of 1,000,000 random natural peptides using the same HLA II.
