# Supplementary material for: Characterization of Conserved and Promiscuous Human Rhinovirus CD4 T Cell Epitopes
Source: Cells. 2021 Sep 2;10(9):2294. doi: 10.3390/cells10092294 (PMC8471592; doi:10.3390/cells10092294)
Supplement: Supplementary file 1 [file cells-10-02294-s001.zip › cells-1341828-supplementary/Table S4.pdf]

Table S4. IFNγ-responses from HLA II-typed donors to RV-specific CD4 T cell epitopes.

Peptide	Sequence	Predicted HLA II binding allele [1]		Confirmed HLA II binding allele [2]		HLA II typing of the responding donors						
		DRB1	DQA1/B1	DRB1	DQA1/B1	Donor	DRB1	DRB1	DQA1	DQA1	DQB1	DQB1
RVA $_{57-74}$	VKDVLEKGIPTLQSPTVE	DRB1*11:01	DQA1*01:02/B1*06:02	-	DQA1*01:02/B1*06:02	#1	07:01	11:01	01:02	03:01	03:03	03:01
						#2	07:01	11:04	02:01	05:01	02:02	03:01
						#3	03:01	11:01	03:01	05:01	02:01	03:01
RVA $_{89-106}$	DSTITSQDVANAVVGYGV	-	DQA1*01:02/B1*06:02<br>DQA1*02:01/B1*02:02<br>DQA1*03:01/B1*03:01<br>DQA1*05:01/B1*03:01	DRB1*04:04<br>**DRB1*07:01**	DQA1*01:02/B1*06:02<br>**DQA1*02:01/B1*02:02**<br>**DQA1*03:01/B1*03:01**<br>DQA1*05:01/B1*02:01<br>**DQA1*05:01/B1*03:01**	#1	07:01	11:01	01:02	03:01	03:03	03:01
						#2	07:01	11:04	02:01	05:01	02:02	03:01
						#3	03:01	11:01	03:01	05:01	02:01	03:01
RVA $_{97-114}$	VANAVVGYGVWPHYLTPE	DRB1*04:04	DQA1*01:01/B1*05:01<br>DQA1*03:01/B1*03:01<br>DQA1*05:01/B1*03:01<br>DQA1*05:01/B1*04:02<br>DQA1*06:01/B1*04:02	DRB1*08:02	DQA1*01:01/B1*05:01<br>**DQA1*05:01/B1*02:01**<br>**DQA1*05:01/B1*03:01**	#1	07:01	11:01	01:02	03:01	03:03	03:01
						#2	07:01	11:04	02:01	05:01	02:02	03:01
						#3	03:01	11:01	03:01	05:01	02:01	03:01
RVC $_{258-274}$	INLRTNNSSTIVVPYIN	DRB1*13:02	DQA1*01:02/B1*05:01<br>DQA1*01:02/B1*06:02<br>DQA1*01:03/B1*06:03<br>DQA1*02:01/B1*03:03<br>DQA1*05:01/B1*03:03	**DRB1*07:01**<br>DRB1*09:01<br>DRB1*13:02	DQA1*01:02/B1*06:02<br>**DQA1*02:01/B1*02:02**	#1	07:01	11:01	01:02	03:01	03:03	03:01
						#2	07:01	11:04	02:01	05:01	02:02	03:01
RVC$_{1582-1592}$	KEKFRDIRRFIP	DRB1*08:02<br>DRB1*11:01	-	DRB1*08:02<br>**DRB1*11:01**<br>**DRB1*11:04**	-	#1	07:01	11:01	01:02	03:01	03:03	03:01
						#2	07:01	11:04	02:01	05:01	02:02	03:01
						#3	03:01	11:01	03:01	05:01	02:01	03:01
RVC $_{1791-1806}$	GLEPLDLNTSAGFPYV	DRB1*07:01<br>DRB1*09:01<br>DRB1*13:02	**-**	DRB1*09:01<br>DRB1*13:02	**DQA1*02:01/B1*02:02**	#1	07:01	11:01	01:02	03:01	03:03	03:01
						#2	07:01	11:04	02:01	05:01	02:02	03:01
						#3	03:01	11:01	03:01	05:01	02:01	03:01
RVC$_{1835-1847}$	DLPYVTYLKDELR	-	DQA1*02:01/B1*02:02<br>DQA1*05:01/B1*02:01	DRB1*04:04	**DQA1*05:01/B1*02:01**	#2	07:01	11:04	02:01	05:01	02:02	03:01
						#3	03:01	11:01	03:01	05:01	02:01	03:01

[1] HLA I molecules predicted to bind the corresponding peptides as retrieved by RANKPEP and NETMHCII servers. [2] HLA II molecule experimentally confirmed to bind the peptide with a high binding affinity (IC_{50} < 1000 nM). HLA II molecules matching any of the alleles expressed by the responding donors are highlighted in bold.
