# Supplementary material for: Characterization of Conserved and Promiscuous Human Rhinovirus CD4 T Cell Epitopes
Source: Cells. 2021 Sep 2;10(9):2294. doi: 10.3390/cells10092294 (PMC8471592; doi:10.3390/cells10092294)
Supplement: Supplementary file 1 [file cells-10-02294-s001.zip › cells-1341828-supplementary/Table_S3.pdf]

**Table S3.** Binding affinity data of RV peptides to HLA II molecules

| Peptide | Sequence | HLA II molecule | IC50 nM | SD |
|---|---|---|---|---|
| RVA 57-74 | VKDVLEKGIPTLQSPTVE | HLA-DQA1*01:01/DQB1*05:01 | 34764 | 1.2 |
| RVA 57-74 | VKDVLEKGIPTLQSPTVE | HLA-DQA1*01:02/DQB1*06:02 | 71 | 1.2 |
| RVA 57-74 | VKDVLEKGIPTLQSPTVE | HLA-DQA1*02:01/DQB1*02:02 | 3079 | 1.1 |
| RVA 57-74 | VKDVLEKGIPTLQSPTVE | HLA-DQA1*05:01/DQB1*02:01 | 27211 | 1.2 |
| RVA 57-74 | VKDVLEKGIPTLQSPTVE | HLA-DQA1*05:01/DQB1*03:01 | 14753 | 1.4 |
| RVA 57-74 | VKDVLEKGIPTLQSPTVE | HLA-DRB1*04:04 | 4103 | 1.2 |
| RVA 57-74 | VKDVLEKGIPTLQSPTVE | HLA-DRB1*08:02 | 38898 | 1.0 |
| RVA 57-74 | VKDVLEKGIPTLQSPTVE | HLA-DRB1*09:01 | 9537 | 1.1 |
| RVA 57-74 | VKDVLEKGIPTLQSPTVE | HLA-DRB1*11:01 | 38843 | 1.0 |
| RVA 89-106 | DSTITSQDVANAVVGYGV | HLA-DQA1*01:01/DQB1*05:01 | 7560 | 2.0 |
| RVA 89-106 | DSTITSQDVANAVVGYGV | HLA-DQA1*01:02/DQB1*06:02 | 33 | 1.2 |
| RVA 89-106 | DSTITSQDVANAVVGYGV | HLA-DQA1*02:01/DQB1*02:02 | 515 | 1.4 |
| RVA 89-106 | DSTITSQDVANAVVGYGV | HLA-DQA1*03:01/DQB1*03:01 | 761 | 1.1 |
| RVA 89-106 | DSTITSQDVANAVVGYGV | HLA-DQA1*05:01/DQB1*02:01 | 907 | 1.1 |
| RVA 89-106 | DSTITSQDVANAVVGYGV | HLA-DQA1*05:01/DQB1*03:01 | 68 | 1.1 |
| RVA 89-106 | DSTITSQDVANAVVGYGV | HLA-DRB1*04:04 | 19 | 2.0 |
| RVA 89-106 | DSTITSQDVANAVVGYGV | HLA-DRB1*07:01 | 865 | 1.2 |
| RVA 89-106 | DSTITSQDVANAVVGYGV | HLA-DRB1*08:02 | 5165 | 1.1 |
| RVA 89-106 | DSTITSQDVANAVVGYGV | HLA-DRB1*09:01 | 1198 | 1.2 |
| RVA 89-106 | DSTITSQDVANAVVGYGV | HLA-DRB1*11:01 | 34921 | 1.2 |
| RVA 89-106 | DSTITSQDVANAVVGYGV | HLA-DRB1*11:04 | 5822 | 1.5 |
| RVA 89-106 | DSTITSQDVANAVVGYGV | HLA-DRB1*13:02 | 6651 | 1.1 |
| RVA 97-114 | VANAVVGYGVWPHYLTPE | HLA-DQA1*01:01/DQB1*05:01 | 20 | 1.3 |
| RVA 97-114 | VANAVVGYGVWPHYLTPE | HLA-DQA1*01:02/DQB1*06:02 | 6407 | 1.3 |
| RVA 97-114 | VANAVVGYGVWPHYLTPE | HLA-DQA1*02:01/DQB1*02:02 | 33942 | 1.2 |
| RVA 97-114 | VANAVVGYGVWPHYLTPE | HLA-DQA1*03:01/DQB1*03:01 | 2254 | 1.1 |
| RVA 97-114 | VANAVVGYGVWPHYLTPE | HLA-DQA1*05:01/DQB1*02:01 | 270 | 1.7 |
| RVA 97-114 | VANAVVGYGVWPHYLTPE | HLA-DQA1*05:01/DQB1*03:01 | 724 | 1.2 |
| RVA 97-114 | VANAVVGYGVWPHYLTPE | HLA-DRB1*04:04 | 5582 | 1.1 |
| RVA 97-114 | VANAVVGYGVWPHYLTPE | HLA-DRB1*07:01 | 1303 | 1.2 |
| RVA 97-114 | VANAVVGYGVWPHYLTPE | HLA-DRB1*08:02 | 453 | 1.3 |
| RVA 97-114 | VANAVVGYGVWPHYLTPE | HLA-DRB1*09:01 | 11527 | 1.1 |
| RVA 97-114 | VANAVVGYGVWPHYLTPE | HLA-DRB1*11:01 | 1100 | 1.3 |
| RVA 97-114 | VANAVVGYGVWPHYLTPE | HLA-DRB1*11:04 | 19653 | 1.0 |
| RVA 97-114 | VANAVVGYGVWPHYLTPE | HLA-DRB1*13:02 | 11594 | 1.1 |
| RVC 258-274 | INLRTNNSSTIVVPYIN | HLA-DQA1*01:01/DQB1*05:01 | 5202 | 2.9 |
| RVC 258-274 | INLRTNNSSTIVVPYIN | HLA-DQA1*01:02/DQB1*06:02 | 67 | 1.4 |
| RVC 258-274 | INLRTNNSSTIVVPYIN | HLA-DQA1*02:01/DQB1*02:02 | 434 | 1.2 |
| RVC 258-274 | INLRTNNSSTIVVPYIN | HLA-DQA1*03:01/DQB1*03:01 | 70047 | 1.0 |
| RVC 258-274 | INLRTNNSSTIVVPYIN | HLA-DQA1*05:01/DQB1*02:01 | 19055 | 2.4 |
| RVC 258-274 | INLRTNNSSTIVVPYIN | HLA-DRB1*04:04 | 3428 | 1.6 |
| RVC 258-274 | INLRTNNSSTIVVPYIN | HLA-DRB1*07:01 | 411 | 1.3 |
| RVC 258-274 | INLRTNNSSTIVVPYIN | HLA-DRB1*09:01 | 840 | 1.0 |
| RVC 258-274 | INLRTNNSSTIVVPYIN | HLA-DRB1*13:02 | 40 | 1.2 |
| RVC 1582-1592 | KEKFRDIRRFIP | HLA-DQA1*01:01/DQB1*05:01 | 2949 | 2.6 |
| RVC 1582-1592 | KEKFRDIRRFIP | HLA-DQA1*02:01/DQB1*02:02 | 33511 | 1.1 |

| | | | | |
|---|---|---|---|---|
| RVC 1582-1592 | KEKFRDIRRFIP | HLA-DQA1*05:01/DQB1*02:01 | 7433 | 1.3 |
| RVC 1582-1592 | KEKFRDIRRFIP | HLA-DRB1*04:04 | 8683 | 1.0 |
| RVC 1582-1592 | KEKFRDIRRFIP | HLA-DRB1*07:01 | 12973 | 1.3 |
| RVC 1582-1592 | KEKFRDIRRFIP | HLA-DRB1*08:02 | 44 | 1.3 |
| RVC 1582-1592 | KEKFRDIRRFIP | HLA-DRB1*09:01 | 2730 | 1.2 |
| RVC 1582-1592 | KEKFRDIRRFIP | HLA-DRB1*11:01 | 33 | 1.1 |
| RVC 1582-1592 | KEKFRDIRRFIP | HLA-DRB1*11:04 | 101 | 1.4 |
| RVC 1791-1806 | GLEPLDLNTSAGFPYV | HLA-DQA1*01:02/DQB1*06:02 | 1920 | 1.1 |
| RVC 1791-1806 | GLEPLDLNTSAGFPYV | HLA-DQA1*02:01/DQB1*02:02 | 191 | 1.3 |
| RVC 1791-1806 | GLEPLDLNTSAGFPYV | HLA-DQA1*03:01/DQB1*03:01 | 10388 | 1.2 |
| RVC 1791-1806 | GLEPLDLNTSAGFPYV | HLA-DQA1*05:01/DQB1*02:01 | 12364 | 1.7 |
| RVC 1791-1806 | GLEPLDLNTSAGFPYV | HLA-DQA1*05:01/DQB1*03:01 | 1817 | 1.4 |
| RVC 1791-1806 | GLEPLDLNTSAGFPYV | HLA-DRB1*03:01 | 8399 | 1.1 |
| RVC 1791-1806 | GLEPLDLNTSAGFPYV | HLA-DRB1*04:04 | 1095 | 1.1 |
| RVC 1791-1806 | GLEPLDLNTSAGFPYV | HLA-DRB1*07:01 | 1198 | 1.1 |
| RVC 1791-1806 | GLEPLDLNTSAGFPYV | HLA-DRB1*09:01 | 7,6 | 1.5 |
| RVC 1791-1806 | GLEPLDLNTSAGFPYV | HLA-DRB1*13:02 | 62 | 1.1 |
| RVC 1835-1847 | DLPYVTYLKDELR | HLA-DQA1*01:01/DQB1*05:01 | 12717 | 3.0 |
| RVC 1835-1847 | DLPYVTYLKDELR | HLA-DQA1*02:01/DQB1*02:02 | 5668 | 1.1 |
| RVC 1835-1847 | DLPYVTYLKDELR | HLA-DQA1*05:01/DQB1*02:01 | 171 | 1.3 |
| RVC 1835-1847 | DLPYVTYLKDELR | HLA-DRB1*04:04 | 773 | 1.4 |
| RVC 1835-1847 | DLPYVTYLKDELR | HLA-DRB1*08:02 | 2596 | 1.2 |
| RVA 57-74 | VKDVLEKGIPTLQSPTVE | HLA-DQA1*03:01/DQB1*03:01 | 70047 | 1.0 |
| RVA 57-74 | VKDVLEKGIPTLQSPTVE | HLA-DRB1*03:01 | 48915 | 1.0 |
| RVA 57-74 | VKDVLEKGIPTLQSPTVE | HLA-DRB1*07:01 | 48915 | 1.0 |
| RVA 57-74 | VKDVLEKGIPTLQSPTVE | HLA-DRB1*11:04 | 48915 | 1.0 |
| RVA 57-74 | VKDVLEKGIPTLQSPTVE | HLA-DRB1*13:02 | 48915 | 1.0 |
| RVA 89-106 | DSTITSQDVANAVVGYGV | HLA-DRB1*03:01 | 48915 | 1.0 |
| RVA 97-114 | VANAVVGYGVWPHYLTPE | HLA-DRB1*03:01 | 48915 | 1.0 |
| RVC 258-274 | INLRTNNSSTIVVPYIN | HLA-DQA1*05:01/DQB1*03:01 | 70047 | 1.0 |
| RVC 258-274 | INLRTNNSSTIVVPYIN | HLA-DRB1*03:01 | 41204 | 1.0 |
| RVC 258-274 | INLRTNNSSTIVVPYIN | HLA-DRB1*08:02 | 40943 | 1.0 |
| RVC 258-274 | INLRTNNSSTIVVPYIN | HLA-DRB1*11:01 | 41204 | 1.0 |
| RVC 258-274 | INLRTNNSSTIVVPYIN | HLA-DRB1*11:04 | 41204 | 1.0 |
| RVC 1582-1592 | KEKFRDIRRFIP | HLA-DQA1*01:02/DQB1*06:02 | 57220 | 1.0 |
| RVC 1582-1592 | KEKFRDIRRFIP | HLA-DQA1*03:01/DQB1*03:01 | 70047 | 1.0 |
| RVC 1582-1592 | KEKFRDIRRFIP | HLA-DQA1*05:01/DQB1*03:01 | 70047 | 1.0 |
| RVC 1582-1592 | KEKFRDIRRFIP | HLA-DRB1*03:01 | 58372 | 1.0 |
| RVC 1582-1592 | KEKFRDIRRFIP | HLA-DRB1*13:02 | 58372 | 1.0 |
| RVC 1791-1806 | GLEPLDLNTSAGFPYV | HLA-DQA1*01:01/DQB1*05:01 | 43779 | 1.0 |
| RVC 1791-1806 | GLEPLDLNTSAGFPYV | HLA-DRB1*08:02 | 43779 | 1.0 |
| RVC 1791-1806 | GLEPLDLNTSAGFPYV | HLA-DRB1*11:01 | 43779 | 1.0 |
| RVC 1791-1806 | GLEPLDLNTSAGFPYV | HLA-DRB1*11:04 | 43779 | 1.0 |
| RVC 1835-1847 | DLPYVTYLKDELR | HLA-DQA1*01:02/DQB1*06:02 | 51989 | 1.1 |
| RVC 1835-1847 | DLPYVTYLKDELR | HLA-DQA1*03:01/DQB1*03:01 | 70047 | 1.0 |
| RVC 1835-1847 | DLPYVTYLKDELR | HLA-DQA1*05:01/DQB1*03:01 | 70047 | 1.0 |
| RVC 1835-1847 | DLPYVTYLKDELR | HLA-DRB1*03:01 | 53882 | 1.0 |
| RVC 1835-1847 | DLPYVTYLKDELR | HLA-DRB1*07:01 | 53882 | 1.0 |

| | | | | |
|---|---|---|---|---|
| RVC 1835-1847 | DLPYVTYLKDELR | HLA-DRB1*09:01 | 53882 | 1.0 |
| RVC 1835-1847 | DLPYVTYLKDELR | HLA-DRB1*11:01 | 53882 | 1.0 |
| RVC 1835-1847 | DLPYVTYLKDELR | HLA-DRB1*11:04 | 53882 | 1.0 |
| RVC 1835-1847 | DLPYVTYLKDELR | HLA-DRB1*13:02 | 53882 | 1.0 |

Table reports mean IC50 values and standard deviations (SD) determined by competitive binding assays using the specified peptides and HLA II molecules. The table is sorted to show at the top the values plotted in Figure 3; the shaded cells at the bottom of the table are values not included in the figure (IC50 > 40000 nM). SD values are geometric.
